# Characterization of a Single-Chain Variable Fragment Recognizing a Linear Epitope of Aβ: A Biotechnical Tool for Studies on Alzheimer’s Disease?

**DOI:** 10.1371/journal.pone.0059820

**Published:** 2013-03-26

**Authors:** Silke Dornieden, Andreas Müller-Schiffmann, Heinrich Sticht, Nan Jiang, Yeliz Cinar, Michael Wördehoff, Carsten Korth, Susanne Aileen Funke, Dieter Willbold

**Affiliations:** 1 Institute of Complex Systems (ICS-6), Forschungszentrum Jülich, Jülich, Germany; 2 Institut für Neuropathologie, Heinrich-Heine-Universität, Düsseldorf, Germany; 3 Institut für Biochemie, Friedrich-Alexander-Universität Erlangen-Nürnberg, Erlangen, Germany; 4 Bioanalytik, Hochschule für Angewandte Wissenschaften, Coburg, Germany; 5 Institut für Physikalische Biologie and BMFZ, Heinrich-Heine-Universität, Düsseldorf, Germany; Cleveland Clnic Foundation, United States of America

## Abstract

Alzheimer’s disease (AD) is a progressive neurodegenerative disorder with devastating effects. Currently, therapeutic options are limited to symptomatic treatment. For more than a decade, research focused on immunotherapy for the causal treatment of AD. However, clinical trials with active immunization using Aβ encountered severe complications, for example meningoencephalitis. Consequently, attention focused on passive immunization using antibodies. As an alternative to large immunoglobulins (IgGs), Aβ binding single-chain variable fragments (scFvs) were used for diagnostic and therapeutic research approaches. scFvs can be expressed in *E. coli* and may provide improved pharmacokinetic properties like increased blood-brain barrier permeability or reduced side-effects in vivo. In this study, we constructed an scFv from an Aβ binding IgG, designated IC16, which binds the N-terminal region of Aβ (Aβ(1-8)). scFv-IC16 was expressed in *E. coli*, purified and characterized with respect to its interaction with different Aβ species and its influence on Aβ fibril formation. We were able to show that scFv-IC16 strongly influenced the aggregation behavior of Aβ and could be applied as an Aβ detection probe for plaque staining in the brains of transgenic AD model mice. The results indicate potential for therapy and diagnosis of AD.

## Introduction

Alzheimer’s disease (AD) is a devastating, progressive, neurodegenerative disorder, which affects more than 35 million people world-wide [1]. Currently, AD treatment is restricted to palliative care due to the lack of disease arresting or modifying therapies [2]. Several lines of evidence have indicated that the amyloid-β-peptide (Aβ) plays a pivotal role in the pathology of AD. Aβ is produced throughout life as a 38 to 43 residue peptide derived from the amyloid precursor protein (APP) after cleavage by two distinct proteases, called β- and γ-secretase [3], [4], [5]. The function of Aβ needs to be clarified, but recent studies suggest neuroprotective effects of monomeric Aβ (for review, see ref. [6]). Senile plaques in the brain of the patient, one of the typical histopathological hallmarks of AD, consist mainly of Aβ(1-42). They are thought to play a crucial role in the pathology of AD, and according to the original amyloid cascade hypothesis, Aβ deposited in plaques has been thought to be responsible for neuronal dysfunction [7], [8]. However, Aβ can adopt a variety of neurotoxic conformers (for review, see ref. [9]), and more recent studies indicate that diffusible Aβ oligomers are the major toxic species during disease development and progression [10], [11], [12], [13]. Consequently, agents that interfere with Aβ oligomerization or increase Aβ clearance from the brain are expected to be valuable for application in therapy or prevention of AD. Passive immunotherapeutic approaches, i.e. direct administration of Aβ antibodies peripherally, were shown to be effective in transgenic mouse models of AD [14], [15], [16], [17]. A variety of humanized monoclonal antibodies are currently investigated in clinical trials (for review, see ref. [18]). However, two phase III clinical trials with antibodies were discontinued as they failed to improve cognitive functions in the treated patients [19].

Additionally, Aβ binding ligands can be valuable for the investigation of the plaque load by *in vivo* imaging methods. Currently, only a few amyloid PET ligands have been applied in clinical studies (for review, see ref. [20], [21]). Numerous efforts are devoted to develop new, target-specific imaging agents for the detection of amyloid plaques *in vivo*. To be suitable, such substances should exhibit highly specific binding to Aβ aggregates, very selective labeling and efficient brain penetration.

Targeting Aβ using scFvs have been shown to be a suitable alternative to IgGs [22]. ScFvs are genetically engineered constructs composed of the variable regions of the heavy-(V_H_) and light chain (V_L_) domains of a respective antibody, connected by a flexible linker to prevent dissociation. They usually retain the specific and monovalent antigenic binding affinity of the parent IgG, but can exhibit improved pharmacokinetic properties like increased blood-brain barrier permeability [23]. Because of their small size, they can be recombinantly expressed in living systems, offering advantages for protein expression in microbial hosts and purification with high yield [24], as well as for gene therapeutic approaches that could avoid repeated infusions of expensive antibodies like performed in passive immunotherapy. Due to the lack of the constant domain (F_C_) in scFvs, F_C_ induced cellular responses, microhemorrhages and inflammatory processes are prevented [25]. These advantages prompted the generation of several Aβ-binding scFvs that interact with Aβ, influence Aβ aggregation and partially reduce Aβ cytotoxicity in cell culture [26], [27], [28]. Recently, adeno-associated virus (AAV)-mediated intramuscular expression of a gene encoding for a scFv against the N-terminal part of Aβ, isolated from a human scFv library [29], was demonstrated to be effective in removing Aβ from the brain of AD transgenic mice without inducing hemorrhages or inflammation. Additionally, the cognitive performance of treated mice was improved in comparison with control mice [30]. Furthermore, another scFv binding to the C-terminus of Aβ, constructed using an antibody that was generated by immunizing mice with Aβ(30-42), has been shown to reduce congiophilic angiopathy as well as plaque burden in APP transgenic mice after direct chronic intranasal treatment [31].

We have constructed a scFv derived from an Aβ binding monoclonal antibody IC16, designated scFv-IC16. ScFv-IC16 was expressed in *E. coli*, purified and characterized with respect to its interaction with different Aβ species and its influence on Aβ fibrilization. Additionally, scFv-IC16 was used to stain and characterize plaques in brain slices of transgenic AD model mice by immunohistochemistry. scFv-IC16 was conclusively found to be a remarkable antibody fragment for the investigation of molecular pathological mechanisms, therapy or diagnosis of AD.

## Materials and Methods

### Molecular Modeling

The structure of IC16 in complex with Aβ(1-8) was generated with Modeller [32] using the crystal structure of the Aβ(1-8)-bound antibody PFA1 as template (pdb code 2IPU; [33]). The model was completed using Sybyl7.3 (Tripos Inc., St. Louis, MO, USA) by addition of Aβ-residue D1, which was not resolved in the PFA-Aβ crystal structure, followed by 100 steps of conjugate gradient energy minimization. The quality of the resulting model was checked using WhatCheck [34] and intermolecular contacts were analyzed with LigPlot [35].

### Materials

All chemicals were supplied by AppliChem GmbH (Darmstadt, Germany), Merck (Darmstadt, Germany), Sigma Aldrich (St. Louis, MO, USA), and Roth (Karlsruhe, Germany) in research grade. All peptides were purchased as reversed phase high performance liquid chromatography purified products (purity > 95%). Synthetic human Aβ(1-42) was purchased from Bachem (Bubendorf, Switzerland). N-terminally biotinylated Aβ(1-42) was purchased from Anaspec (Fremont, CA, USA). Aβ peptides Aβ(1-8)-GSGSC, Aβ(2-8)-GSGSC, Aβ(3-8)-GSGSC, and Aβ(8-15)-GSGSC were purchased from JPT (Berlin, Germany).

### Construction of an IC16 Single-chain Variable Fragment (scFv-IC16) Expression Plasmid

The monoclonal IgG2a antibody IC16 recognizes Aβ(1-8) and has been described earlier [36]. Briefly, an IC16 hybridoma was generated by standard fusion procedure of myeloma cells with splenocytes from a PrP knockout mouse [37] immunized with KLH Aβ(1-16). A scFv was constructed with mRNA purified from IC16 hybridoma used for PCR amplification with primers specific for variable regions of the heavy (V_H_) and light chain (V_L_): V_H_ forward: 5′- AAAACCATGGCGCAGGTTACTCTGAAAGAGTC-3′; V_H_ reverse: 5′ TTTTGCCGGCCAG TGGATAGACCGATGGGGCTGTTGTTTTGGT-3′; V_L_ forward: 5′ AAAAGGATCCGATGTTTTGATGACCCAAACT-3′;V_L_ reverse: 5- AAAAGCGGCCGCGGATACAGTTGGTGCAGCATC-3′. PCR products were digested with *Ngo*MIV (V_H_) or *Bam*HI (V_L_) and ligated to a oligonucleotide coding for a (Gly_4_Ser)_3_ linker domain [38]. The resulting 800 bp product was eluted from agarose gel and amplified with V_H_ forward and V_L_ reverse primer. The amplificate was digested with *Nco*I and *Eag*I and ligated into pET22b (Merck, Darmstadt, Germany), allowing periplasmic expression of the IC16-scFv fused to a His_6_- and Myc-tag. The complete amino acid sequence of scFv-IC16 is as follows: MKYLLPTAAAGLLLLAAQPAMAMAQVTLKESGPGILQPSQTLSLTCSFSGFSLSTSGMGV.

SWIRQPSGKGLEWLAHIFWDDDKNYNPSLKSRLTVSKDTSRNQVFLKITSVDTSDTATYY.

CARSPHLRGYDVDFDYWGQGTTLTVSSAKTTAPSVYPLAGGGGSGGGGSGGGGSDVLMTQ.

TPLSLPVSLGDQASISCRSSQSLVHSNGNTYLHWYLQKPGQSPKVLIYKVSNRFSGVPDR.

FSGSGSGTDFTLKISRVEAEDLGVYFCSQSTHVPLTFGAGTKLELKRADAAPTVSAAEEQ.

KLISEEDLEEEEEEGTLEHHHHHH.

### Generation of Aβ(1-16)-GB1-NHS-Sepharose for Purification of scFv-IC16

For expression of Aβ(1-16)-GB1 (Aβ-GB1), human Aβ(1-16) was ligated into the *Nde*1 site of pET22b-GB1 [39]. Aβ-GB1 was expressed at 37°C in BL21(DE3) Rosetta (Merck, Darmstadt, Germany) grown to mid-logarithmic phase in 2YT-medium. Expression was induced by the addition of IPTG to a final concentration of 1 mM, and cells were then grown for a further 4 h. After harvesting cells by centrifugation, they were lysed in 50 mM Tris-HCl (pH 8.0), 5 mM EDTA, 1% Triton X-100, 2 mM phenylmethanesulfonyl fluoride (PMSF), 20 mM MgCl_2_, 200 U DNase1 and 0,25 mg/ml lysozyme. The cleared lysate was first dialyzed to 20 mM Tris-HCl (pH 8.0) and 1 mM EDTA and the first purification step was then performed via Q-Sepharose (GE Healthcare, Little Chalfont, UK). The column was washed with dialysis buffer and bound Aβ-GB1 was eluted with 20 mM Tris-HCl (pH 8.0), 1 mM EDTA and 75 mM NaCl. In a final purification step, Aβ-GB1 was separated from minor impurities using IgG-Sepharose (GE Healthcare). After washing with 20 mM Tris-HCl (pH 8.0), 150 mM NaCl and 1 mM EDTA, Aβ-GB1 was eluted with 50 mM glycine pH 2.5 and immediately neutralized by adding Tris-HCl (pH 8.0) to a final concentration on 100 mM. After dialysis against PBS the protein was coupled to NHS-Sepharose (GE Healthcare, Little Chalfont, UK) according to the manufacturer’s recommendations.

### Expression and Purification of scFv-IC16

*Escherichia coli* strain BL21(DE3)pRARE2 was used as an expression host. Bacteria were grown to high density (OD600≥1.6) at 37°C, and cooled on ice for 1 h before induction with IPTG at a final concentration of 0.1 mM. Cells were further incubated for 24 h at 18°C, and subsequently harvested by centrifuging for 30 min at room temperature at 5000×g. Cells were lysed in 20 mM Tris-HCl pH 8.0, 0.4 mM EDTA, 5 mM imidazole, 500 mM NaCl, 20 mM MgCl_2_, 10 mM CaCl_2_, Protease Inhibitor Cocktail Tablet Complete EDTA free (Roche, Grenzach-Wyhlen, Germany), 1 mg/ml lysozyme and 500 U DNase. The lysate was cleared by centrifugation at 20 000×g, and the soluble protein in the supernatant was purified via Ni-NTA chromatography (Ni-NTA Agarose, AppliChem GmbH, Darmstadt, Germany). After loading the sample onto the Ni-NTA Agarose, the column (3 ml) was washed with 10 column volumes (CV) 20 mM Tris-HCl pH 8.0, 5 mM imidazole, 500 mM NaCl and 1% TX-100, followed by a second wash with 10 CV 20 mM Tris-HCl pH 8.0, 5 mM imidazole, 500 mM NaCl. Bound scFv-IC16 was eluted by four CV elution buffer (20 mM Tris-HCl pH 8.0, 300 mM imidazole, and 300 mM NaCl). A second purification step using affinity chromatography was performed, because the purity of the eluted protein was lower than 50%. The previously generated Aβ1-16-GB1 NHS sepharose was used for a subsequent purification step. After loading scFv-IC16 (in elution buffer), the column was washed with 10 CV TBS (137 mM NaCl, 2.7 mM KCl, 2.5 mM Tris-HCl, pH 7.4). The protein was eluted with 50 mM glycine, pH 2.5 and immediately neutralized with a final concentration of 100 mM Tris-HCl, pH 8.0. ScFv-IC16 was dialyzed against PBS (137 mM NaCl, 2.7 mM KCl, 1.8 mM KH_2_PO_4,_ 10 mM Na_2_HPO_4_, pH 7.4) and stored at −20°C until further usage.

### Binding Constant Determination Using Surface Plasmon Resonance (SPR)

Binding kinetics were determined by SPR using a Biacore™ X (GE Healthcare, UK). Synthetic Aβ peptides were dissolved in 10 mM NaAc, pH 4.0. The CM5 sensor chip surface (GE Healthcare, UK) was activated using N-ethyl-N′-3 (diethylaminopropyl) carbodiimide (EDC) and N-hydroxysuccinimide (NHS) chemistry followed by 2-(2-pyridinyldithio)ethaneamine hydrochloride (PDEA), in order to introduce a reactive thiol group. Aβ was coupled via the C-terminal cysteine to the chip at a flow rate of 5 µl/min, and the remaining active groups were blocked by injecting cysteine. The immobilization procedure was performed according to manufacturer’s recommendation. All kinetic analyses were performed at a flow rate of 20 µl/min in PBS. Varying concentrations of scFv-IC16 (10 to 5000 nM) and IC16 (10 to 1000 nM) were injected. Association was observed for 180 s whereas the dissociation was observed for 120 to 360 s. When required, the surface was regenerated by injecting 20 µl 50 mM glycine, pH 11.0. The data evaluation was performed using Biaevaluation Software 4.1.1. ScFv-IC16 data were fitted according to the Langmuir 1∶1 binding model, whereas IC16 data were fitted according to the bivalent binding model. Standard errors of equilibrium dissociation constants (K_D_) were calculated using standard errors of the corresponding association and dissociation rate constants [40].

### Thioflavin T (ThT) Assays

7.5 µM Aβ(1-42) (stock solution of 190 µM in DMSO, freshly prepared before usage) were incubated with 10 µM ThT in PBS in absence or presence of different concentrations of scFv-IC16 (1.5, 3.75 and 7.5 µM) at room temperature for 24 h. In addition, scFv-IC16 (7.5 µM) without Aβ(1-42) was tested under the same conditions as the negative control. For all samples a reference value, generated with PBS and ThT only, was subtracted. Each sample had a volume of 50 µl and was incubated and measured in a 384 well plate (Greiner 384 Well polypropylene Greiner Bio-One GmbH, Frickenhausen, Germany). The plate was covered by an adhesive film preventing evaporation of the sample buffer. Fluorescence recordings were performed every 30 min for 22 h (excitation wavelength 440 nm, emission wavelength 490 nm, Polarstar Optima, BMG, Ortenberg, Germany). The experiments were performed five-fold, and the average of the obtained values as well as the standard deviation was calculated.

### Seedless-preparation of Aβ1-42

1 mg of Aβ(1-42) or N-terminally/C-terminally biotinylated Aβ(1-42) was dissolved in 1 ml 1,1,1,3,3,3-hexafluorosiopropanol (HFIP) and incubated overnight at room temperature. Afterwards, Aβ(1-42) was mixed in the favored ratio with N-terminally/C-terminally biotinylated Aβ(1-42) and aliquoted. HFIP was evaporated using a SpeedVac (Concentrator 5301, Eppendorf, Germany) at room temperature for 30 min. To evaporate remaining HFIP, the sample was incubated overnight at room temperature with an open lid.

### Preparation of Aβ(1-42) Monomers, Oligomers and Fibrils

The preparation of Aβ(1-42) monomers and oligomers was carried out by size exclusion chromatography (SEC) as already described by Johansson et al [41] with minor modifications. 250 µg of Aβ peptide was dissolved in 130 µl SEC buffer (50 mM NaPi, pH 7.4; 150 mM NaCl; 0.6% Tween-20) and briefly centrifuged (1 min, 16 100×g, room temperature) to remove insoluble fibrillar material. 100 µl of the supernatant was applied on a Superdex75 10/300 column (GE Healthcare, UK) connected to an Äkta purifier system (GE Healthcare, UK). The sample was eluted at a flow rate of 0.6 ml per min at room temperature and detected at wavelengths of 214, 250 and 278 nm. Fractions of 200 µl were collected. For the preparation of Aβ fibrils, 125 µg of Aβ(1-42) peptide was dissolved in 200 µl PBS and incubated for 24 h (300×g, 37°C). Samples were centrifuged (16 100×g, 20 min, room temperature) and the supernatant containing soluble Aβ species was discarded. Insoluble fibrils were resuspended in 200 µl SEC buffer. The concentration of the Aβ preparations was determined using the Micro BCA Protein Assay Kit (Thermo Scientific, Waltham, MA, USA). Before performing the standard protocol recommended by the manufacturer, 80 µl 6 M urea was mixed with 80 µl monomer- and oligomer preparation, respectively. As the concentration of the fibril preparation was usually high, 80 µl 6 M Urea was mixed with 40 µl SEC buffer and 40 µl fibril fraction. The samples were incubated at 60°C for 30 min. Afterwards they were mixed with BCA reagent at a ratio of one to one and again incubated at 60°C for 20 to 30 min. Bovine serum albumin (BSA) at concentrations of 10, 20 and 40 µg per ml was used as a standard. The samples were measured at a wavelength of 570 nm with a Polarstar Optima plate reader (BMG, Ortenberg, Germany).

### Enzyme-linked Immunosorbent Assay (ELISA) Analysis of scFv-IC16 Binding to Aβ-monomers, -oligomers and -fibrils

An ELISA was applied to characterize the binding affinity of scFv-IC16 to different Aβ conformers. After concentration determination of the Aβ(1-42) monomer, oligomer and fibril preparations, each conformer was diluted in immobilization buffer (0.1 M NaHCO_3_, pH 9.3) to a concentration of 5 µg/ml, and coated on Nunc immobilizer streptavidin F96 clear plates (250 ng/well) (Nunc, Thermo Scientific, Waltham, MA, USA). Alternatively, Aβ monomers were prepared seedless in the same concentration by dissolving 50 µg N-terminally biotinylated Aβ(1-42) in 1 ml immobilization buffer and diluting it further to 5 µg/ml. N- or C-terminally biotinylated Aβ(1-10) peptides were dissolved in immobilization buffer to a final concentration of 1.2 µg/ml (60 ng/well). 50 µl of these Aβ solutions were incubated in the well for 1 h. Each well was washed with 100 µl immobilization buffer and the plate was stored at 4°C overnight in 100 µl immobilization buffer per well. Unspecific binding sites were blocked with 50 µl blocking buffer (PBS, 1% BSA) at room temperature for 1 h. After removal of the blocking buffer, a solution of either scFv-IC16 (12 and 23 nM in PBS-T (0.1% Tween-20) and 0.1% BSA respectively), IC16 (0.67 nM PBS-T and 0.1% BSA) or mAB-6E10 (0.67 nM PBS-T and 0.1% BSA) was added to each conformer and incubated for 1 h. Subsequently, each well was washed with PBS-T (three times, 100 µl) and incubated with Penta-His antibody (Qiagen, Hilden, Germany, dilution 1∶500, in 2 ml TBS-T) for 1 h at room temperature. This was followed by another three washing steps with PBS-T and detection of the antibodies with HRP-conjugated anti-mouse IgG (Thermo Scientific, Waltham, MA, USA, dilution 1∶10000, in 2 ml TBS-T). 3,3,5,5-tetramethylbenzidine (Sigma Aldrich, St. Louis, MO, USA) was used as the substrate for the HRP. The reaction was terminated by adding sulfuric acid to a final concentration of 1 M. The absorbance was recorded at 450 nm on Polarstar Optima plate reader (BMG, Ortenberg, Germany).

### Immunoprecipitation of Cell Culture Derived Aβ

Purified scFv-IC16 was dialyzed against PBS and coupled to NHS-Sepharose (GE, Buckinghamshire, UK) according to the manufacturers recommendations. Conditioned medium (CM) was prepared from confluent wild type CHO cells or 7PA2 cells cultured in DMEM (Life Technologies, UK) in presence or absence of FCS. Aβ was immune-precipitated from cleared CM with scFv-IC16-NHS-Sepharose overnight at 4°C. After washing with PBS, captured proteins were separated by 10–20% tricine peptide PAGE (Biorad, Hercules, CA) and transferred onto a 0.2 µm nitrocellulose membrane at 400 mA for 2 h. The filter was boiled for 5 min in PBS [42] and blocked overnight at 4°C with 5% fat-free milk in PBS containing 0.05% Tween 20 (PBS-T). After washing in PBS-T, the membrane was probed with 1∶200 diluted monoclonal 4G8 (Signet, Dedhem, MA). Bound antibody was detected with horseradish peroxidase conjugated goat anti-mouse Ig (at 1∶25000) (Thermo Scientific, Bonn, Germany). Subsequently, the Amersham ECL Western Blotting Detection Reagent (GE, Buckinghamshire, UK) was applied for visualization.

### Ex vivo Staining of Plaques in Mouse Brain Slices

Deep frozen horizontal brain cryosections (20 µm thickness) from 9 or 12 months old Tg2576 mice and the respective wildtype control were dried in ambient air for 15 min, and then fixed in ice-cold 4% (w/v) paraformaldehyde for 20 min. Incubation with 70% formic acid for 5 min was performed to enhance immunoreactivity. Sections were permeabilized with 1% Triton X-100 in TBS for 10 min, followed by blocking with Mouse on mouse (M.O.M.) Basic Kit (Vector Laboratories) in TBS-Triton (1%, v/v) for 30 min. Primary antibodies were diluted in 1% BSA/TBS-Triton (working concentration of ScFv: 400 nM, 6E10∶33 nM, 1-11-3 (Covance, USA) in 1∶200 dilution) and probed on the sections for 1 h, incubating at room temperature. For double staining of scFv-IC16 and 1-11-3, the respective antibodies were mixed directly in 1% BSA/TBS-Triton buffer. Subsequently, the slices were incubated overnight at 8°C in a humid chamber. For His-tagged scFv, sections were incubated with anti Penta-His Mouse antibody (Qiagen) 1∶10 diluted in 1% BSA/TBST for 2 h at room temperature. A secondary antibody (Goat anti Mouse Alexa 488, Invitrogen), diluted 1∶300 in 1% BSA/TBST, was incubated for 2 hours at room temperature. For double staining, two secondary antibodies (goat anti mouse-Alexa488 and goat anti rabbit-Alexa-568 were mixed directly (working concentration of goat anti rabbit-Alexa568∶1.300). After 1 min of incubation with 0.5 µg/ml DAPI, the sections were coverslip-mounted with Aqua Poly/Mont (Polysciences Inc, Worrington, PA, USA). Sections were examined under a fluorescence microscope (Leica LMD6000 (Leica, Solms, Germany). Excitation Range: UV; blue; green. Excitation Filter: Bp 420/30; Bp 495/15; Bp 570/20) with camera (Leica DFC 310 FX). Images were processed with LAS software (Leica Application Suite, V.4.0.0) and ImageJ (1.45 s).

## Results

### Expression of scFv-IC16 in *E. coli* and Purification via Ni-NTA and Aβ-affinity Chromatography

**Figure 1:**
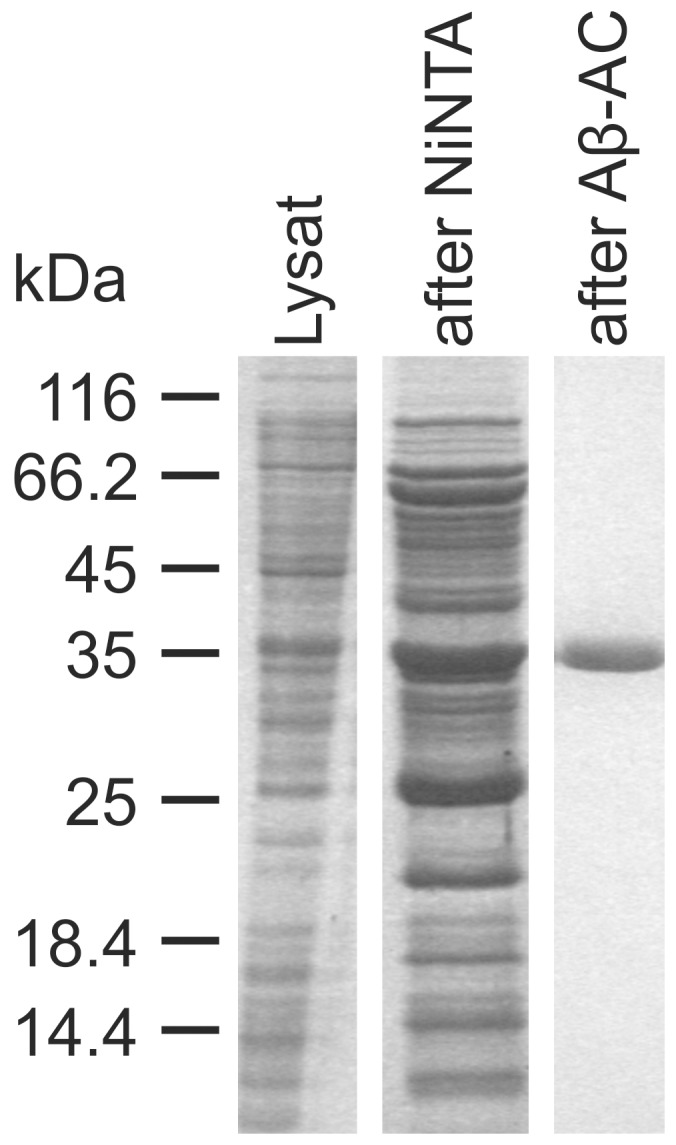
SDS-PAGE analysis of the purification of scFv-IC16 via Ni-NTA and Aβ1-16 GB1 NHS sepharose affinity chromatography. 1 µl of the lysate and 15 µl of the purified fractions were applied to 16% polyacrylamide gels [56]. Lane 1: cell lysate, lane 2: eluate after affinity chromatography via Ni NTA–Agarose, lane 3: eluate after Aβ1-16-GB1-NHS-Sepharose affinity chromatography. Target protein is more than 99% pure after final purification step.

To characterize scFv-IC16, the protein was produced in *E. coli* and purified via Ni-NTA and Aβ-affinity chromatography. Expression was performed using the BL21DE3 pRARE2 strain. ScFv expression often results in formation of inclusion bodies [43], [44]. Therefore, we have developed a protocol for slow expression, leading to an increase of correctly folded and soluble protein. Cells were shocked on ice for 1 h after reaching the stationary phase (OD_600_≥1.6) in order to slow down the metabolism. Protein expression was induced with a final IPTG concentration of 0.1 mM. Under these conditions more than 10% of the expressed protein was soluble. Attempts to purify the remaining unsoluble protein after denaturation were successful and yielded pure protein after Ni-NTA affinity chromatography. However, refolding attempts remained unsuccessful. Purification of natively folded protein was performed using Ni-NTA affinity chromatography, followed by Aβ affinity chromatography using Aβ(1-16)-GB1 NHS Sepharose. IC16 was originally raised against Aβ(1-16). Therefore, the fusion protein contains the binding epitope of scFv-IC16. In addition, this step ensured that only active and correctly folded scFv was purified and thus used for further studies. The complete procedure resulted in 0.4±0.1 mg of pure protein as judged by SDS-PAGE (Fig. 1) and UV/Vis spectrometry, derived from 1 L cell culture.

### Characterization of the Binding Site of scFv-IC16 at Aβ(1-16) – Homology Modeling and Surface Plasmon Resonance Measurements

**Figure 2:**
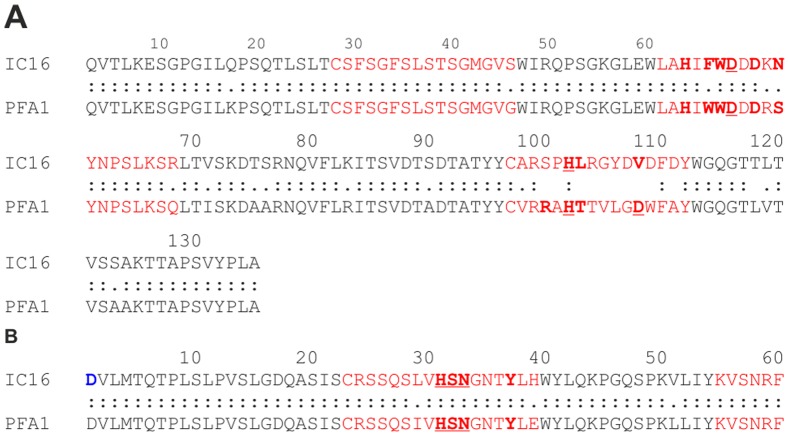
Sequence alignment of the V_H_ (a) and V_L_ (b) region of IC16 and PFA1. Residues belonging to the CDRs are marked in red. Labeling was done according to the sequence of the heavy and light chain used for the scFv-IC16 construct, each starting with residue 1. Residues that interact with Aβ(1-8) are marked in bold and those residues that form hydrogen bonds or salt-bridges with Aβ(1-8) are additionally underlined. D1 of the IC16 light chain is marked in bold and blue, because it forms repulsive interactions with D1 of Aβ.

**Figure 3:**
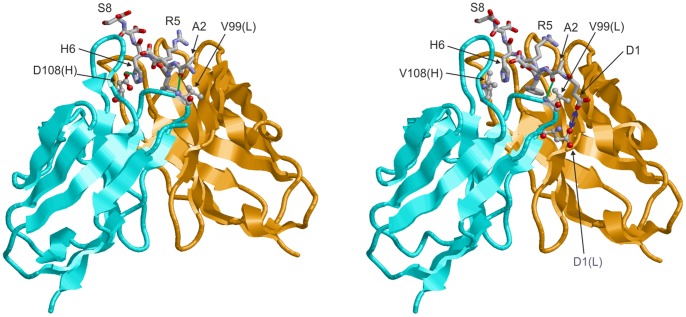
Interaction of monomeric Aβ(1-8) with the antibodies PFA1 (left) and IC16 (right). Residues of Aβ(1-8) are shown in stick presentation and colored according to the atom type (residue D1 of Aβ is not resolved in the PFA1 complex). For clarity, only D1, A2, R5, H6, and S8 of Aβ(1-8) are labeled. Key interacting residues of the antibodies are shown in ball-and-stick presentation and are labeled (chain identifier in parenthesis). Polar attractive interactions formed by these residues are depicted in green line. A repulsive interaction between D1(L) and D1 of Aβ(1-8) is indicated by a blue double-headed arrow.

The amino acid sequence of the constructed scFv-IC16 was compared to antibody fragments that were already described in the literature. The IgG2a monoclonal anti-Aβ protofibril antibodies PFA1 and PFA2 were derived from mice challenged with a stabilized protofibril form of Aβ(1-40). The derived Fab fragments exhibit binding to Aβ monomers in the nM range, but the binding to aggregated Aβ forms is significantly impaired in comparison to the full IgG molecules. Structural characterization of the Fabs in complex with the Aβ(1-8) peptide as well as binding studies revealed a significant influence of N-terminal Aβ truncations on binding [33]. Our alignments exhibited sequence identities of 82% and 94% for the V_H_ and V_L_ regions of scFv-IC16 and the Fab fragment PFA1, respectively (Fig. 2, [33]). In addition, the complementary determining regions (CDR), which are hypervariable and mainly determine the binding properties of an antibody [45], showed high similarity of scFv-IC16 to PFA1 (see also Tables 1 and 2). Therefore, we assume that the binding of scFv-IC16 is similar to Aβ as shown for PFA1 and Aβ(1-8) by X-ray crystallography. To understand the binding of scFv-IC16 to Aβ and the differences to the binding of PFA1 to Aβ in more detail, the structure of scFv-IC16 in complex with Aβ(1-8) was modeled using the crystal structure of the Aβ(1-8)-PFA1 complex [33] as a template. As expected from the high degree of sequence similarity, both antibodies form similar contacts with Aβ(1-8) (Fig. 3). Differences are only detected for sequence positions 100 and 108 of the heavy chain, as well as 98 of the light chain. Of these three sites, only residue 108 forms significant interactions with Aβ(1-8). In PFA1, D108(H) forms a salt-bridge with H6 of Aβ, while V108(H) of IC16 forms nonpolar interactions with H6 (Fig. 3).

**Table 1:** Comparison of the CDR sequences of the heavy chains of different antibody fragments.

Name	CDR1	CDR2	CDR3
**scFv-IC16**	GFSLSTSGMGV	HIFWDDDKNYNPSLKSR	RSPHLRGYDVDFDY
**PFA1**	GFSLSTSGMG	IWWDDDR	VRRAHTTVLGDWFAY
**PFA2**	GFSLRTSGMG	IWWDDDK	VRRAHNVVLGDWFAY

**Table 2:** Comparison of the CDR sequences of the light chains of antibody fragments.

Name	CDR1	CDR2	CDR3
**scFv-IC16**	RSSQSLVHSNGNTYLH	YKVSNRFS	SQSTHVPLT
**PFA1**	QSIVHSNGNTY	KVS	FQGSHVPLTS
**PFA2**	QSIVHSNGNTY	KVS	FQGSHVPLTS

**Figure 4:**
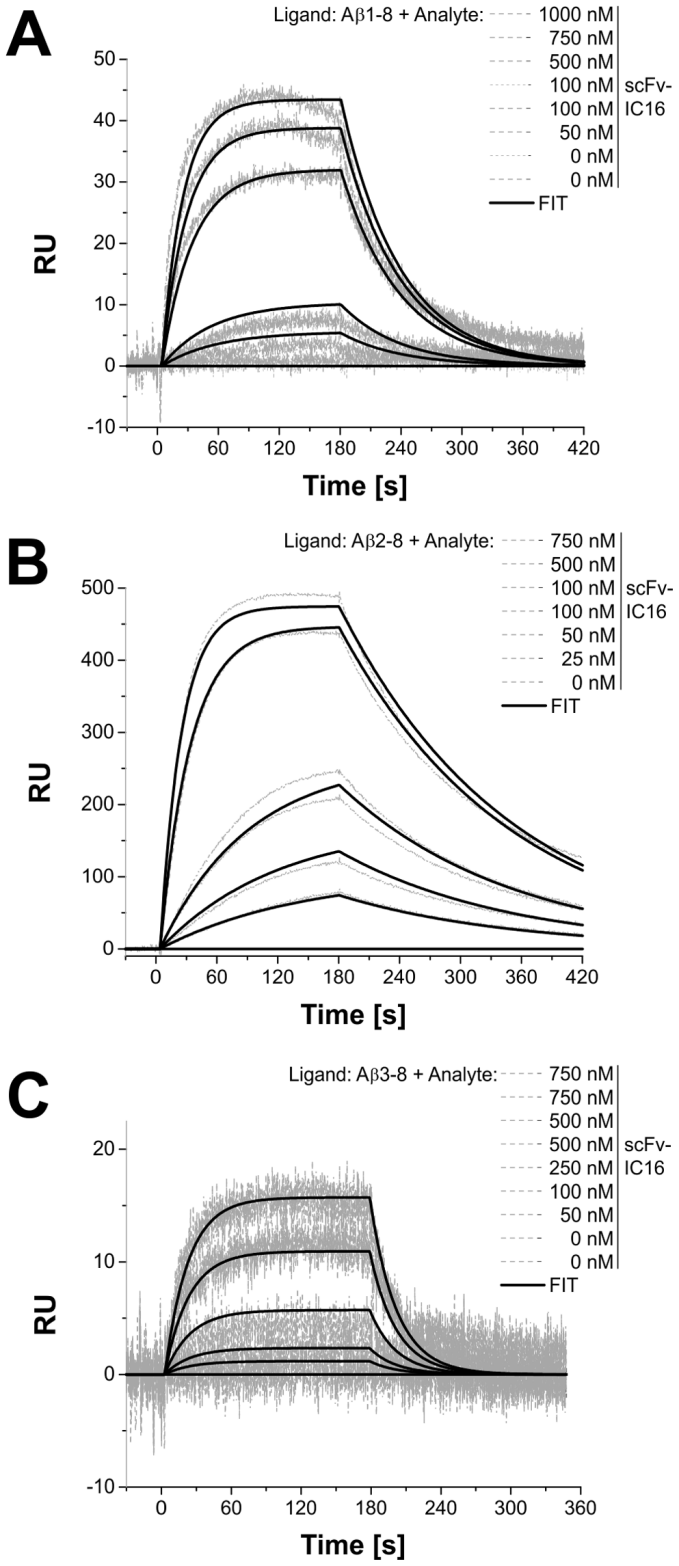
Analysis of the binding affinity of scFv-IC16 to immobilized Aβ peptides. (A: Aβ(1-8), B: Aβ(2-8) and C: Aβ(3-8)) using SPR. Depicted are the overlaid sensorgrams of different injection concentrations of scFv-IC16 onto an Aβ peptide coupled CM5 sensor chip. Each injection was performed for 3 min at a flow rate of 20 µl/min. Concentrations from 0 to 1000 nM were injected (see legend) in consecutive order. Dissociation was observed 2 to 4 min following an injection. Selected concentrations were injected twice as controls. The experimental data were fitted using the Langmuir 1∶1 binding model (Ri = 0). [25°C; Running buffer: PBS, RmaxAβ(1-8) = 67.5; RmaxAβ(2-8) = 553; RmaxAβ(3-8) = 126].

**Figure 5:**
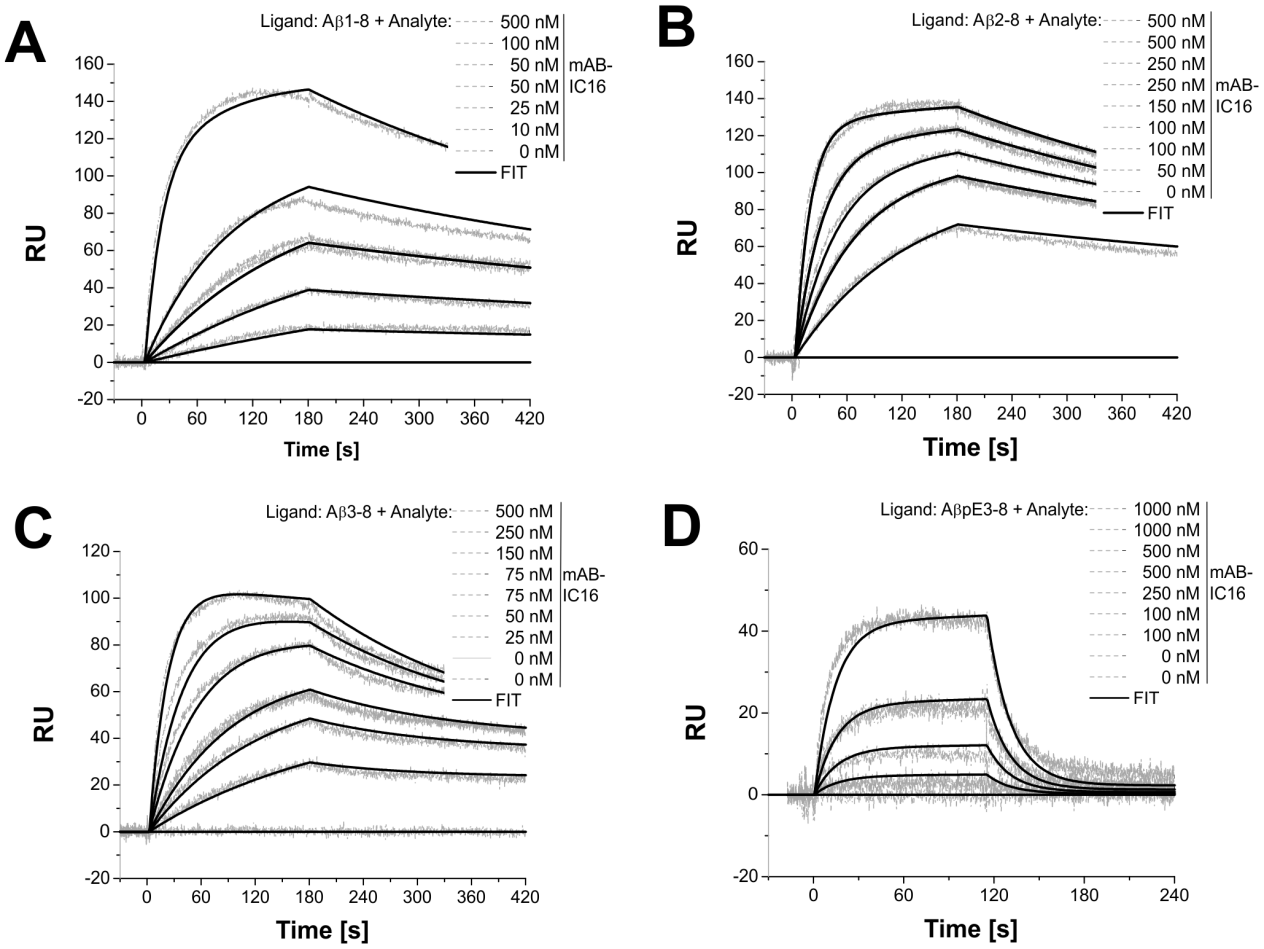
Analysis of the binding affinity of IC16 to immobilized Aβ peptides. (A: Aβ(1-8), B: Aβ(2-8), C: Aβ(3-8) and D: Aβ(pE3-8)) using SPR. Depicted are the overlaid sensorgrams of different injection concentrations of IC16 onto an Aβ peptide coupled CM5 sensor chip. Each injection was performed for 3 min at a flow rate of 20 µl/min. Concentrations from 0 to 1000 nM were injected (see legend) in consecutive order. Dissociation was observed for 2 to 4 min. Selected concentrations were injected twice as additional controls. The experimental data were fitted using the bivalent binding model (Ri = 0). [25°C; Running buffer: PBS; RmaxAβ(1-8) = 169; RmaxAβ(2-8) = 154; RmaxAβ(3-8) = 121; R_max_Aβ(pE3-8) = 360].

Our next aim was to confirm the prediction of the modeling. Consequently, the equilibrium constants (K_D_) of scFv-IC16 to various N-terminal Aβ fragments were determined using SPR. For comparison, we also performed the measurements with the monoclonal parent antibody IC16 (Fig. 4 and 5, Table 3). The monoclonal antibody was expected to exhibit more structural stability caused by the constant domains. Additionally, an avidity effect may influence the binding constants of IC16 to the different Aβ peptides. Measurements on both Aβ(1-8) and Aβ(8-15) were used to confirm the localization of the linear binding epitope at the N-terminal. No interaction of IC16 and scFv-IC16 with Aβ(8-15) was observed. For Aβ(1-8) the injection curves showed a fast association rate of IC16 and a very slow dissociation rate. This result is reflected in the low K_D_ of 51 nM (Table 3, bivalent fit). Comparison of the binding curves of IC16 and scFv-IC16 revealed that the association rates of scFv-IC16 and IC16 to Aβ(1-8) were comparable, whereas the dissociation rate of scFv-IC16 was one order of magnitude higher than the corresponding IC16 dissociation rate. These results are reflected in the K_D_ for scFv-IC16 (K_D_ of 0.55 µM, Table 3, Langmuir 1∶1 binding model), demonstrating a loss of affinity of the scFv in comparison to the monoclonal antibody, which may be due to the lost avidity of the scFv as compared to the monoclonal antibody.

**Table 3:** Rate constants and dissociation constants of the interactions of scFv-IC16 and IC16 with N-terminal Aβ fragments.

Analyte	Ligand	*k*_on_ (M^-1^ s^-1^ )	*k*_off_ (s^-1^)	*K*_D_ (M)
**scFv-IC16**	Aβ(1-8)	3.10±0.02×10^4^	1.72±0.005×10^−2^	553±4×10^−9^
	Aβ(2-8)	6.84±0.03×10^4^	7.65±0.03×10^−3^	112±0.6×10^−9^
	Aβ(3-8)	8.36±0.5×10^3^	43.9±0.3×10^−3^	5.25±0.3×10^−6^
	Aβ(pE3-8)			n.d.
	Aβ(8-15)			n.d.
**IC16**	Aβ(1-8)	3.5±0.02×10^4^	1.8±0.02×10^−3^	51±0.5×10^−9^
	Aβ(2-8)	4.85±0.01×10^4^	1.54±0.006×10^−3^	32±0.1×10^−9^
	Aβ(3-8)	4.17±0.006×10^4^	3.43±0.007×10^−3^	82±0.2×10^−9^
	Aβ(pE3-8)	4.21±0.008×10^4^	63±0.05×10^−3^	1.5±0.001×10^−6^
	Aβ(8-15)			n.d.

Data were fitted globally. scFv-IC16 data were fitted according to the Langmuir 1∶1 binding model, whereas IC16 data were fitted using a bivalent fit. The association rate constants (k_on_), the dissociation rate constants (k_off_) and the equilibrium dissociation constants (K_D_) are given below. Both, scFv-IC16 and IC16, did not show any detectable binding towards Aβ(8-15). However, they showed binding to all N-terminal peptides, except scFv-IC16 did not bind to Aβ(pE3-8). The lowest K_D_ value was observed for the interaction of scFv-IC16 and IC16 to Aβ(2-8).

A comparison of the affinities of both IC16 and scFv-IC16 for N-terminally truncated Aβ-peptides (Table 3) showed that the highest affinities were observed for Aβ(2-8). Deletion of A2 leading to Aβ(3-8) resulted in significant reduction of affinity for scFv-IC16 (Table 3). Truncation of the two N-terminal residues and modification of E3 into pyroE3 led to an extensive loss of affinity and no binding could be detected for scFv-IC16.

### Influence of scFv-IC16 on Aβ(1-42) Fibrilization

**Figure 6:**
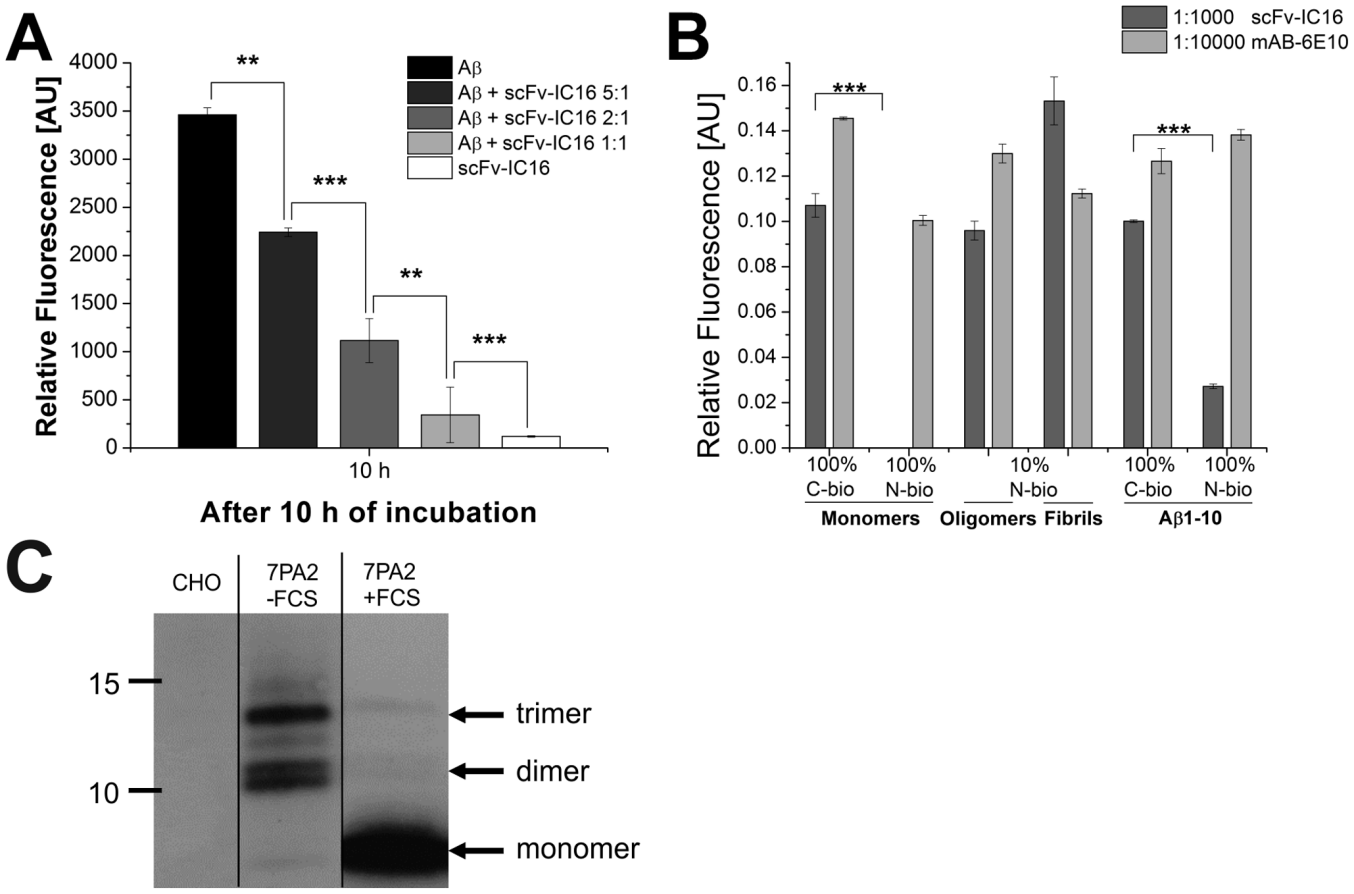
Inhibition of ThT positive Aβ fibril formation in presence of different scFv-IC16 concentrations. (A). ScFv-IC16 was added in concentrations of: 7.5, 3.75, 1.5 and 0 µM (positive control) to 7.5 µM Aβ(1-42) samples. Data were recorded every 30 min during 22 hours of incubation at room temperature. Depicted is the absolute fluorescence value after 10 h of incubation. Upon addition of ThT, fluorescence was measured at 490 nm in relative units (mean +/− standard deviations of results, each measurement was repeated five times). All values are corrected by background fluorescence of ThT in PBS. ScFv-IC16 only control (7.5 µM) does not show any formation of ThT positive fibrils. A statistical significant difference between the fluorescence values of Aβ only and Aβ-scFv-IC16 co incubations was calculated by student’s T-test, as indicated (**: p<0.01; ***: p<0.001). Analysis of an ELISA experiment to quantify the binding specificity of scFv-IC16 to different Aβ1-42 conformers and Aβ1-10 peptides (B). 250 ng Aβ1-42 (100% N-biotinylated monomers, 100% C-terminally biotinylated monomers, 10% N-biotinylated oligomers and fibrils) and 60 ng Aβ peptides 1–10 (100% C-terminally biotinylated Aβ1-10, 100% N-terminally biotinylated Aβ1-10) were immobilized on streptavidin coated 96 well plates. 6E10 was used to monitor the immobilized amount of Aβ conformers. Similar absorption values of 6E10 to the different Aβ conformers except for 100% N-terminally biotinylated monomers, indicate similar molar amounts of immobilized Aβ regarding monomeric Aβ for monomers, oligomers and fibrils. Background absorption was subtracted from all samples. A highly significant difference in the relative fluorescence value between N-biotinylated monomers and C-biotinylated monomers, as well as between C-bio Aβ1-10 and N-bio Aβ1-10 was calculated by Student’s t-test (***: p<0.001). N-bio = N-terminally biotinylated; C-bio = C-terminally biotinylated. Western Blot of Aβ monomers and low-n oligomers immunoprecipitated by scFv-IC16 from conditioned medium (CM) of CHO or 7PA2 cells (C). The latter were grown either in medium with or without FCS. Monomeric Aβ is stabilized by the presence of FCS, whereas low-n oligomeric Aβ is eriched in CM-FCS. No Aβ was precititated from the supernatants of CHO cells. Monomeric Aβ (CM 7Pa2+ FCS) and low-n oligomeric Aβ (CM 7PA2− FCS) were equally bound by scFv-IC16. Detection antibody: 4G8.

To characterize the influence of scFv-IC16 on Aβ(1-42) fibrilization, ThioflavinT (ThT) assays were carried out. ThT is a benzothiazole dye, which exhibits a shift and increase in quantum yield while binding to β-sheet rich fibrils [46]. During the fibrilization process of Aβ, an increase of ThT fluorescence can be observed until a saturation level is reached. Whereas the Aβ sample showed a strong increase in ThT fluorescence after 10 h, ThT assays with Aβ and scFv-IC16 yielded a scFv-IC16 dose-dependent lower ThT signal (Fig. 6A), indicating a strong influence of scFv-IC16 on Aβ fibril formation. With the ratio of 1∶1 for Aβ(1-42) and scFv-IC16 the fibrilization of Aβ(1-42) was completely inhibited. At the ratios of 2∶1 and 5∶1, scFv-IC16 reduced the ThT fluorescence significantly. The sample that contained only scFv-IC16 was used as a negative control and did not show ThT fluorescence after 10 h.

### ELISA Analysis of the Binding Activities of scFv-IC16 to Aβ Monomers, Oligomers and Fibrils

The monoclonal antibody IC16 was raised against Aβ(1-16), and binding activity of scFv-IC16 to N-terminal Aβ fragments was confirmed using SPR. Nonetheless, we intended to analyze the binding activities of scFv-IC16 to different Aβ conformers in order to detect potential binding preferences. To perform ELISA studies, Aβ monomers, oligomers and fibrils were prepared and equal molar amounts with respect to monomeric Aβ were immobilized in wells of a 96 well plate. The commercially available monoclonal antibody 6E10 is known to bind to all Aβ conformers with similar affinities as it recognizes human Aβ(3-10) epitope, which is freely accessible in Aβ monomers as well as in oligomers and fibrils [47]. It is frequently used as a standard in ELISA experiments [48], [49], [50] and was used here as a control to compare the immobilized amounts of Aβ monomers, oligomers and fibrils. The different Aβ conformers were incubated with scFv-IC16. Subsequently, bound scFv-IC16 was detected using an anti-His antibody that was then detected by an HRP conjugated secondary antibody. Fig. 6B depicts the relative quantification of the binding of scFv-IC16 and 6E10 to different Aβ conformers. The 6E10 control demonstrated the presence of similar amounts of Aβ monomers, oligomers and fibrils with respect to monomeric Aβ in the wells of the microtiterplate. Furthermore, scFv-IC16 showed a preferential binding to Aβ fibrils. The highest relative fluorescence was detected for fibril binding; the relative fluorescence for oligomer binding was weaker (p = 0.0014, N-bio oligomers to N-bio fibrils). Signals for monomer binding were not detected if Aβ monomers were immobilized onto the streptavidin functionalized surface of the well via an N-terminal biotin tag, indicating interference of the N-terminal biotin tag with the binding of scFv-IC16 to 100% N-terminally biotinylated monomers. Aβ monomers or Aβ(1-10) fragments that were immobilized via a C-terminal biotin tag were bound by scFv-IC16 very similar to Aβ oligomers in the ELISA experiment, which can be shown by similar absorption values (p = 0.038, C-bio monomers to N-bio oligomers; p = 0.19, C-bio Aβ1-10 to N-bio oligomers).

To confirm that scFv-IC16 also binds Aβ that was naturally secreted by 7PA2 cells, scFv-IC16 was coupled to NHS-Sepharose and incubated with culture medium derived from wildtype CHO cells or 7PA2 cells. 7PA2 cells secrete high amounts of monomeric and lower oligomeric Aβ species. Previously it was demonstrated that serum stabilizes the monomeric form of Aβ, whereas oligomeric Aβ accumulates in the absence of serum [51]. As demonstrated in Fig. 6C, scFv-IC16 efficiently precipitated monomeric Aβ from 7PA2-CM containing FCS as well as lower oligomeric Aβ species from CM of 7PA2 cells cultured without FCS. Thus, scFv-IC16 displays the same binding specificity as the monoclonal antibody IC16 [36].

### Ex vivo Staining of Plaques in Mouse Brain Slices

**Figure 7:**
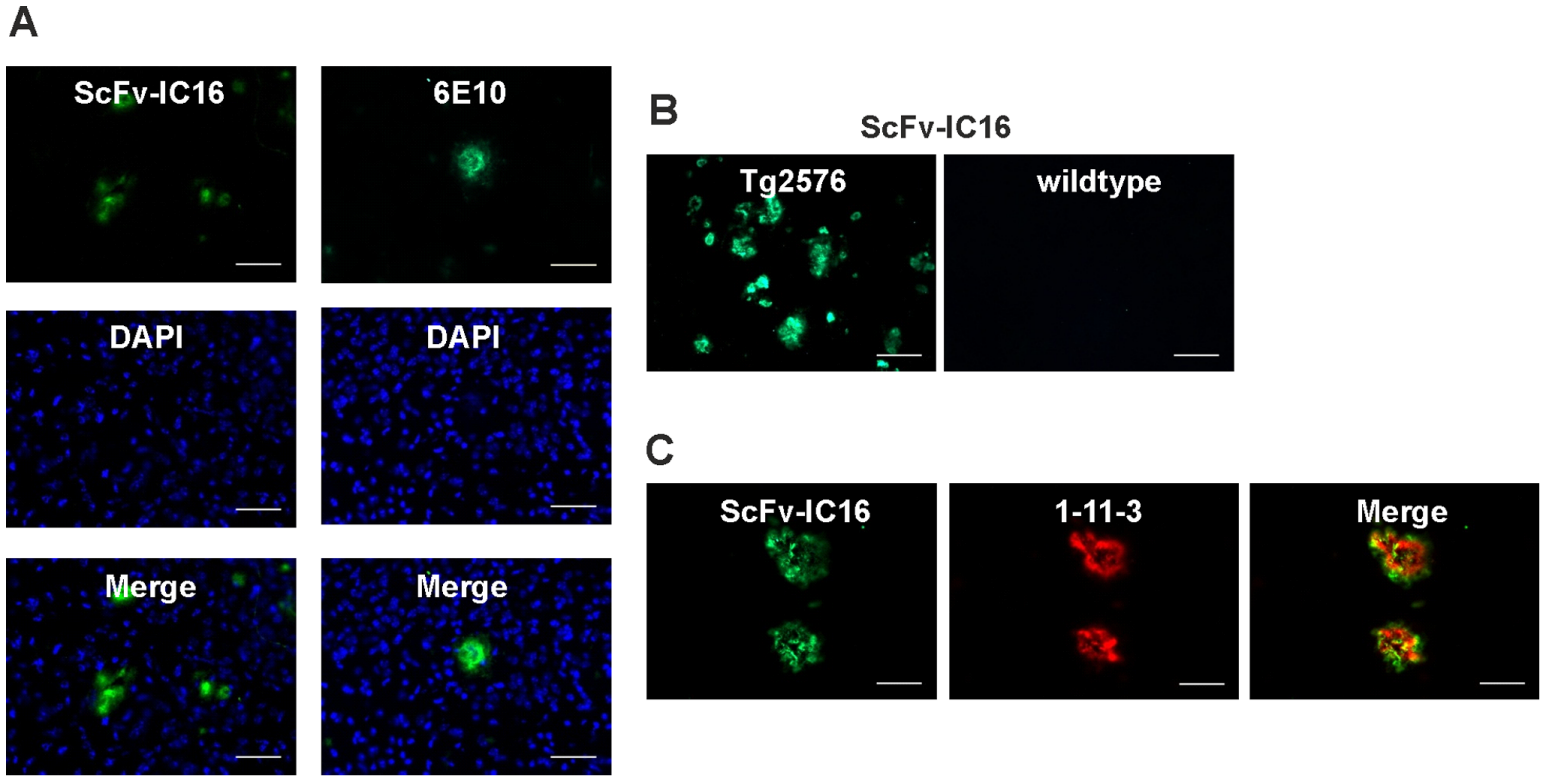
In vitro staining of brain sliced from transgenic 2576 AD mice using scFv-IC16, 6E10-Aβ-antibody and DAPI. (A). Pretreated deep frozen horizontal brain cryosections (20 µm thickness) from 9 months old Tg2576 mice were fixed in 4% paraformaldehyde, treated with 70%formic acid and either incubated with scFv-IC16 in a concentration of 400 nM or 6E10 in a concentration of 33 nM. As scFv-IC16 is fused with a His tag, the respective sections were incubated with Penta-His Mouse antibody. Detection was performed using goat anti mouse-Alexa488 using a fluorescent microscope (Leica LMD6000. Excitation Range: UV; blue; green. Excitation Filter: Bp 420/30; Bp 495/15; Bp 570/20) with camera (Leica DFC 310 FX). Images were processed with LAS software (Leica Application Suite, V.4.0.0) and ImageJ (1.45 s). Scale bar: 50 µm. B: In vitro staining of brain sliced from transgenic 2576 AD mice using scFv-IC16 and a respective wildtype control mouse. Scale bar:100 µm. C: Co-staining of brain sliced from transgenic 2576 AD mice using scFv-IC16 and anti Aβ(1-42) 1-11-3. As secondary antibodies, a mix of goat anti mouse-Alexa488 and goat anti rabbit-Alexa-568 was used. Scale bar: 50 µm.

Brain cryosections derived from nine or 12 months old transgenic (tg 2576) mice and the respective non-transgenic control were stained using scFv-IC16. Additionally, anti-Aβ plaque stainings (using the 6E10 antibody) were performed on adjacent sections as well as costainings with scv-IC16 and 1-11-3 anti Aβ(1-42) antibody. DAPI nuclei counterstainings were performed on the same slides, respectively. Photomicrographs of the stained slices and overlay images are shown in Figure 7. scFv-IC16 could readily be used to detect plaques specifically in AD transgenic mice with low background signals, demonstrating potential applicability as a molecular probe. The staining pattern of scFv-IC-16 was very similar to the staining pattern of 6E10, which also binds to the N-terminal part of Aβ and the periphery of the plaques. The staining pattern of 1-11-3 is slightly different as the core of the plaques is stained with higher intensity.

## Discussion

Here we have characterized a novel scFv with respect to its binding specificities to different Aβ conformers and its influence on Aβ fibrilization. This scFv-IC16 was constructed from an IgG2a, designated IC16, which was raised in mouse against the antigen Aβ(1-16) and binds to Aβ(2-8) [36].

scFv-IC16 shares 87% sequence identity with of PFA1 and PFA2 (see Figure 2), two Aβ binding Fab fragments described by Gardberg et al. [33]. Especially, the three CDRs of scFv-IC16 are highly similar (see Tables 2 and 3), CDRs 1 of the heavy chain are 100% identical and CDRs 1 and CDRs 2 of the light chain are 91% and 100% identical, respectively. The striking WWDDD motif within CDR2 of PFA1 and PFA2 that recognizes the Aβ binding motif EFRH (Aβ(3-6)) [33], [52] is almost completely present in scFv-IC16, too: FWDDD. Because of the high sequence homology between scFv-IC16 and PFA1, we assumed similar interactions between scFv-IC16 and Aβ as described for PFA1 and Aβ. Therefore, a homology modeling for the scFv-IC16-Aβ complex based on the X-ray structure of the Fab PFA1 and Aβ(1-8) was performed (Fig. 3). The model yielded similar binding modes for scFv-IC16 to Aβ(1-8) and PFA1 to Aβ(1-8). However, amino acid D1 of Aβ was not resolved in the X-ray structure of the Aβ(1-8)/PFA1 complex. Our homology modeling placed it in close spatial proximity with the amino acid D1 of the light chain. This close spatial proximity may generate a repulsive force and thus explains the increased affinity (K_D_ ∼112 nM) of scFv-IC16 to Aβ(2-8) as compared to Aβ(1-8) (Tab. 3). The model furthermore offers an explanation why the K_D_ rises to more than 5 µM when the second amino acid A2 of Aβ is truncated. A2 forms a hydrogen bond with the backbone amide group of V99 of the heavy chain and is therefore important for a tight binding. Deletion of residues one and two and modification of Aβ E3 into pyroE3, as found in several truncated and more aggregation prone Aβ species in the diseased brain [53], [54], showed a decrease in affinity and no binding was observed for scFV-IC16.

In comparison, PFA 1 shows the strongest binding to Aβ(1-40) monomers (K_D_∼39 nM). The binding to Aβ(2-7) is in the same order of magnitude (K_D_∼60 nM). Similar to scFv-IC16, the binding of PFA1 to Aβ(pE3-8) is decreased, which is reflected in the K_D_ of ∼3 µM [33], [52]. pE3 lacks the E3 sidechain carboxyl group that forms two hydrogen bonds with S32(H) in the complex crystal structure.

Due to the high sequence similarity between PFA1 and scFv-IC16, similar interactions between scFv-IC16 and E3 of Aβ are also present in our modeled complex structure. Therefore, a loss of the interactions described above may lead to a loss in affinity, so that an interaction between scFv-IC16 and Aβ(pE3-8) could not be detected.

Altogether, the binding of scFv-IC16 to the Aβ fragments is weaker in comparison to PFA1. This may be due to small differences in the binding pocket like the D108V replacement (Fig. 3), but also to the fact that PFA1 is a Fab fragment, consisting of the variable and one constant domain of the heavy chain and the variable and the constant domain of the light chain. The two constant domains may provide more stability for the binding pocket of PFA1 compared with the binding pocket of scFv-IC16, because its two variable domains are connected only via a peptide linker lacking the stability of the two constant domains.

In a series of ELISA experiments, the specificity of scFv-IC16 to different conformers of Aβ, monomers, oligomers and fibrils, was characterized. The non-conformation specific monoclonal antibody 6E10 was used to control the relative amount of bound Aβ conformer in the wells. 6E10 was previously characterized to bind an Aβ N-terminal epitope at residues 3 to 10. Similar ELISA readouts for 6E10 and Aβ monomers, oligomers and fibrils proved that all three conformers were loaded in similar amounts into the wells. The binding of scFv-IC16 to its epitope at residues 2–8 of Aβ monomers that were immobilized using an N-terminal biotin tag was sterically hindered. As soon as the N-terminus was freely accessible, e.g. in C-terminally biotinylated Aβ monomers, scFv-IC16 bound Aβ monomers with the same affinity as oligomers, as judged by the ELISA results. A slightly higher ELISA signal was detected for the binding to Aβ fibrils. Given the fact that scFv-IC16 binds to residues 2–8 of the N-terminus of Aβ, specificity for a special Aβ conformer was not expected. In aggregated Aβ conformers, the N-terminus of Aβ (Aβ(1-9)) was described to be disordered and accessible, whereas residues 10–22 and 30–40 adopt a β-strand conformation and are involved in fibrilization [55]. As both, scFv-IC16 and 6E10, bind to the N-terminus of Aβ, a similar binding pattern after usage for ex-vivo plaque staining in AD transgenic mice was expected. A significantly higher concentration of scFv-IC16 was needed to perform proper plaque staining in comparison to the monoclonal antibody 6E10. The need of higher amounts of protein, however, can be balanced by the fact that the scFv can be expressed in *E. coli* and produced in reasonable amounts at low costs. An interesting option could also be to enhance the affinity of scFv-IC16 to Aβ by rational design or directed evolution methods. In conclusion, scFv-IC16 has several interesting features. It can be produced recombinantly, a fact that also provides possibilities for sequence optimization. scFv-IC16 prevents the formation of ThT positive Aβ(1-42) fibrils. It recognizes all conformers of Aβ as it binds to a linear N-terminal epitope. Furthermore, it is suitable as a molecular probe, which was demonstrated by ex vivo plaque staining in brain slices of AD transgenic mice. Therefore, scFv-IC16 is interesting for therapeutic, imaging and mechanistic studies.
